# Biomechanics of Cell Membrane

**DOI:** 10.3390/ijms21155413

**Published:** 2020-07-30

**Authors:** Stefano Leporatti, José L. Toca-Herrera

**Affiliations:** 1 CNR Nanotec-Istituto di Nanotecnologia c\o Campus Ekotecne Via Monteroni, 73100 Lecce, Italy; 2 Department of Nanobiotechnology, Institute for Biophysics, Muthgasse 11 (Simon Zeisel Haus), University for Natural Resources and Life Sciences Vienna (BOKU), A-1190 Vienna, Austria; jose.toca-herrera@boku.ac.at

This Special Issue is focused on measuring and characterizing the mechanical and adhesive properties of cells and membranes.

Cell mechanics has an important impact on cellular functions and cell fate. The field has developed very rapidly in the last two decades, incorporating elements of cell biology, biophysics and nanotechnology. In particular, the study of lipid membranes, erythrocytes, and endothelial cells have been performed with several techniques, such as scanning force microscopy, laser pinzette, optical tweezers, and many others. Although a great advance has been achieved in recent years, the characterization of the cells properties and the understanding of the results at cellular and molecular level are still a major challenge for researchers in this area.

In this issue, the principles and techniques used in studies of cell and membrane biomechanics and adhesion have been addressed. The reader will find five comprehensive experimental research articles in the oncological field, and another article dealing with cell membrane adhesion modelling, which reports the last developments in the area of adhesion and biomechanics of cancer cells. Experimentally, three of them are closely connected, since the authors used a combination of atomic force microscopy and confocal and/or fluorescence microscopy to investigate morpho-structural/cytomechanical modifications induced by anti-neoplastic drugs or proteins in cancer cells and/or the cell membrane.

In one of these contributions, Mariafrancesca Cascione and co-workers [1], in an attempt to better define the involvement of TGF-β1 in the metastatic progression process in different hepatocarcinoma cell lines (HepG2, PLC/PRF/5, HLE), applied a systematic morphomechanical approach, in order to investigate the physical and the structural characteristics, and evaluated the antitumor effect of LY2157299 (Galunisertib), a TGF-βR1 kinase inhibitor, from a biomechanical point of view, by using atomic force and confocal microscopy. Since the epithelial mesenchymal transition (EMT) is a physiological multistep process involving epithelial cells acquiring a mesenchymal-like phenotype, it was demonstrated that it is linked to tumor progression and metastasis. The transforming growth factor (TGF)-β pathways have been widely investigated, but its role in the hepatocarcinoma EMT is still unclear. The authors [1] found that the epithelial cell exhibited a more elastic behaviour after TGF-β1, suggesting increased migratory capability. On the contrary, in mesenchymal cells, they demonstrated an opposite effect after Galunisertib treatment. These results envisaged the development of antimetastatic HCC therapies based on the inhibition of TGF-β1 receptors and suggested that the use of their approach as new diagnostic tool to be combined with standard biomolecular techniques.

On the same line, Jagoba Iturri et al. [2] employed Atomic force microscopy (AFM) combined with fluorescence microscopy, to quantify cytomechanical modifications induced by resveratrol (at a fixed concentration of 50 μM) in a breast cancer cell line (MCF-7), upon temporal variation. They quantified Young’s modulus, the maximum adhesion force, and tether formation, and thereby, they determined important insight into cell motility and adhesiveness. Going into more detail, by AFM cell indentation measurements, they could determine simultaneous variations in factors such as the Young’s modulus, the maximum adhesion force, and the stepwise zero-force recovery (tether formation), factors that play a critical role in terms of cell motility and stickiness. Moreover, time dependence fits well into three distinct levels (short incubation, 24, and 48 h) according to the impact observed. Maintenance of a drug incubation for longer periods (48 h) induced a gradual loss of properties that concludes in cell death [2]. These results confirmed the validity of the AFM technique as a decisive tool to detect irreversible transformations at the nanoscale level, that might affect normal cell functioning, or even their malignancy, as in the case of tumoral cells during the early steps of drug treatment.

In another paper with a similar approach, Xiaoli Zhang and co-authors [3] have investigated the role of ezrin phosphorylation and its intracellular localization on cell motility, cytoskeleton organization, and cell stiffness, using fluorescence live-cell imaging, image quantification, and atomic force microscopy (AFM). They showed that cells expressing constitutively active ezrin T567D (phosphomimetic) migrate faster and in a more directional manner, especially when ezrin accumulates at the cell rear. Furthermore, image quantification results revealed that transfection with ezrin T567D alters the cell’s gross morphology and decreases cortical stiffness. On the other hand, constitutively inactive ezrin T567A accumulates around the nucleus, leading to a significant buildup of actin fibers, a decrease in nuclear volume, and an increase in cytoskeletal stiffness [3]. Noteworthy cell transfection with the dominant negative ezrin FERM domain has induced significant morphological and nuclear changes and affects actin, microtubules, and the intermediate filament vimentin, resulting in cytoskeletal fibers that are longer, thicker, and more aligned. The authors proposed that the overexpression of phosphorylated ezrin was associated with a reorganization of the cellular cytoskeleton, leading to a decrease in the cortical stiffness, and an increase in the cytoskeleton stiffness. Furthermore, they highlighted the importance of phosphorylated ezrin as a biomarker for cancer metastasis diagnosis, and envisaged ezrin phosphorylation as a promising molecular target for cancer therapy, especially to suppress cancer invasion and metastasis [3].

In an interesting study, Egor Pavlenko and co-workers [4] characterized point mutations within the *ADAM17* gene found in the tissue of colon cancer patients. In order to shed light on the role of ADAM17 in cancer development, as well as into the mechanisms that regulate maturation and cellular trafficking of ADAM17, they perform overexpression investigations of four ADAM17 variants located in the pro-, membrane-proximal- and cytoplasmic-domain of the ADAM17 protein in ADAM10/17-deficient HEK cells. They found a cancer-associated point mutation within the pro-domain of ADAM17 (R177C), to be most impaired in its proteolytic activity and trafficking to the cell membrane, and discovered similar functional limitations [4]. Finally, they proposed the crucial importance of the pro-domain on enzyme maturation and function and suggested ADAM17 as a modulator in colon cancer by its importance in inflammatory events, mediating tumorigenesis. Future studies evaluating ADAM17 function in colon cancer development and how interference with ADAM17 function might be beneficial will necessarily be performed in the respective animal models.

Another contribution authored by Jutamas Uttagomol et al. [5] investigated the impact of cyclic strain and substrate stiffness on Dsg3 expression, and its role in mechanotransduction in keratinocytes. A direct comparison was made with E-cadherin, a well-characterized mechanosensor. Exposure of oral and skin keratinocytes to equiaxial cyclic strain promoted changes in the expression and localization of junction assembly proteins. This study demonstrated that Dsg3 regulated the expression and localization of yes-associated protein (YAP), a mechanosensory, and an effector of the Hippo pathway [5]. Moreover, they showed that Dsg3 formed a complex with phospho-YAP and sequestered it to the plasma membrane, while Dsg3 depletion had an impact on both YAP and phospho-YAP in their response to mechanical forces, increasing the sensitivity of keratinocytes to the strain or substrate rigidity-induced nuclear relocation of YAP and phospho-YAP. Finally, they further demonstrated that this Dsg3/YAP pathway had an influence on the expression of *YAP1* target genes and cell proliferation [5]. The current study collectively underscored the importance of desmosomes as mechanosensory and load-bearing structures that coordinate with AJs in control of tissue integrity and homeostasis.

In the only theoretical contribution, Long Li et al. [6] proposed a mechanical model for catch-bond-mediated cell adhesion in shear flow. The stochastic reaction of bond formation and dissociation was described as a Markovian process, whereas the dynamic motion of cells followed classical analytical mechanics. The steady state of cells significantly depended on the shear rate of flow. The upper and lower critical shear rates required for cell detachment and attachment are extracted, respectively [6]. When the shear rate increased from the lower to the upper threshold, cell rolling became slower and more regular, implying the flow-enhanced adhesion phenomenon. These studies suggest that this flow-enhanced stability of rolling adhesion is attributed to the competition between stochastic reactions of bonds and the dynamics of cell rolling, instead of force lengthening the lifetime of catch bonds, thereby challenging the current view in understanding the mechanism behind this flow-enhanced adhesion phenomenon [6]. These results will help one to understand the mechanical mechanism of catch-bond-mediated rolling adhesion of cells in answer to hydrodynamic impact and can envisage the design of target therapy in biomedical applications.

Finally, we are confident that these articles will readily contribute to the progress in the area of nano/biomechanics, and will further stimulate future studies dedicated to understanding the link between the mechanical properties of cells and membranes, as well as the physico-biological mechanisms and pathways involved.
